# Supplementary material for: Structural insights into the activation and inhibition of CXC chemokine receptor 3
Source: Nat Struct Mol Biol. 2024 Jan 4;31(4):610–20. doi: 10.1038/s41594-023-01175-5 (PMC11026165; doi:10.1038/s41594-023-01175-5)
Supplement: Supplementary file 1 — Supplementary Tables 1 (Sequence of synthesized CXCR3 and CXCL11 genes used for protein expression in Sf9 cells) and 2 (Primers for the generation of CXCR3 and its mutants used in cAMP assay). [file 41594_2023_1175_MOESM1_ESM.pdf]

nature structural & molecular biology

Article

https://doi.org/10.1038/s41594-023-01175-5

Structural insights into the activation and inhibition of CXC chemokine receptor 3

In the format provided by the authors and unedited

Supplementary Table 1. Sequence of synthesized CXCR3 and CXCL11 genes used for protein expression in *Sf9* cells.

Bril-CXCR3-LgBit (in pFastBac1)

ATGAAGACTATCATCGCTCTGTCCTACATCTTCTGCCTGGTGTTCGCTGACTACAAGGACGATGAC
GACAAGGGTTCCGCTGACCTGGAGGACAACTGGGAGACTCTGAACGACAACCTGAAGGTGATCGA
GAAGGCTGACAACGCTGCTCAAGTGAAGGACGCTCTGACTAAGATGAGAGCTGCCGCTCTGGACG
CTCAGAAGGCTACTCCACCAAAGCTGGAGGACAAGTCCCCTGACTCCCCTGAGATGAAGGACTTC
AGACACGGTTTCGACATCCTGGTGGGTCAGATCGACGACGCTCTGAAGCTGGCTAACGAGGGTAA
GGTGAAGGAGGCTCAAGCTGCTGCCGAGCAGTTGAAGACTACTAGAAACGCTTACATTCAGAAGTA
CCTGCTGGTGCCAAGAGGTTCCATGGTGCTGGAGGTGTCCGACCACCAAGTGCTGAACGACGCTG
AGGTGGCTGCTCTGCTGGAGAACTTCTCCTCTTCCTACGACTACGGTGAGAACGAGTCCGACTCCT
GCTGCACTTCCCCACCATGCCCACAAGACTTCTCCCTGAACTTCGACAGAGCTTTCCTGCCTGCTC
TGTACTCCCTGCTGTTCCTGCTGGGTCTGCTGGGTAACGGTGCTGTGGCTGCTGTGCTGCTGTCTA
GAAGAACTGCTCTGTCCTCCACTGACACTTTCCTGCTGCACCTGGCTGTGGCTGACACTCTGCTGG
TGCTGACTCTGCCACTGTGGGCTGTGGACGCTGCTGTGCAGTGGGTGTTCGGTTCCGGTCTGTGC
AAGGTGGCTGGTGCTTTGTTCAACATCAACTTCTACGCTGGTGCTCTGTTGCTGGCTTGCATCTCCT
TCGACAGATACCTGAACATCGTGCACGCTACTCAGCTGTACAGAAGAGGTCCACCTGCTAGAGTGA
CTCTGACTTGCCTGGCTGTGTGGGGTCTGTGCCTGCTGTTCGCTCTGCCTGACTTCATCTTCCTGT
CCGCTCACCACGACGAGAGACTGAACGCTACTCACTGTCAGTACAACTTCCCACAAGTGGGTCGC
ACTGCTCTGAGAGTGCTGCAGCTGGTGGCTGGTTTCCTGCTGCCACTGCTGGTGATGGCTTACTG
CTACGCTCACATCCTGGCTGTGTTGCTGGTCTCTAGAGGTCAGAGAAGACTGAGAGCTATGAGACT
GGTGGTCGTGGTCGTGGTGGCTTTTGCTCTGTGCTGGACTCCATACCACCTGGTCGTGCTGGTGG
ACATCCTGATGGACCTGGGTGCTCTGGCTAGAAACTGCGGTAGAGAGTCTAGAGTGGACGTGGCT
AAGTCCGTGACTTCCGGTCTGGGTTACATGCACTGCTGCCTGAACCCACTGCTGTACGCTTTCGTG
GGTGTGAAGTTCAGAGAGAGAATGTGGATGTTGCTGCTGCGCCTGGGTTGCCCAAATCAGAGAGG
TCTGCAGAGACAGCCATCCTCTTCTAGAAGAGACTCCTCCTGGTCCGAGACTTCCGTGTTCACTCT
GGAGGACTTCGTGGGTGACTGGGAGCAGACTGCTGCTTACAACCTGGACCAAGTGCTGGAGCAAG
GTGGTGTGTCCTCCCTGCTGCAGAACCTGGCTGTGTCCGTGACTCCAATTCAGAGAATCGTGAGAT
CCGGTGAGAACGCTCTGAAGATCGACATCCACGTGATCATCCCATACGAGGGTCTGTCTGCTGATC
AGATGGCTCAGATCGAAGAGGTGTTCAAGGTGGTGTACCCTGTGGACGACCACCACTTCAAGGTG
ATCCTGCCATACGGTACTCTGGTGATCGACGGTGTGACTCCAAACATGCTGAACTACTTCGGTAGA
CCTTACGAGGGTATCGCTGTGTTCGACGGTAAGAAGATCACTGTGACTGGTACTCTGTGGAACGGT
AACAAGATCATCGACGAGCGCCTGATCACTCCTGACGGTTCCATGCTGTTCAGAGTGACTATCAAC
TCCTAA

Bril-CXCR3$^{\kappa OR}$ (in pFastBac1)

ATGAAGACTATCATCGCTCTGTCCTACATCTTCTGCCTGGTGTTCGCTGACTACAAGGACGATGAC
GACAAGGGTTCCGCTGACCTGGAGGACAACTGGGAGACTCTGAACGACAACCTGAAGGTGATCGA
GAAGGCTGACAACGCTGCTCAAGTGAAGGACGCTCTGACTAAGATGAGAGCTGCCGCTCTGGACG
CTCAGAAGGCTACTCCACCAAAGCTGGAGGACAAGTCCCCTGACTCCCCTGAGATGAAGGACTTC
AGACACGGTTTCGACATCCTGGTGGGTCAGATCGACGACGCTCTGAAGCTGGCTAACGAGGGTAA
GGTGAAGGAGGCTCAAGCTGCTGCCGAGCAGTTGAAGACTACTAGAAACGCTTACATTCAGAAGTA
CCTGCTGGTGCCAAGAGGTTCCATGGTGCTGGAGGTGTCCGACCACCAAGTGCTGAACGACGCTG
AGGTGGCTGCTCTGCTGGAGAACTTCTCCTCTTCCTACGACTACGGTGAGAACGAGTCCGACTCCT
GCTGCACTTCCCCACCATGCCCACAAGACTTCTCCCTGAACTTCGACAGAGCTTTCCTGCCTGCTC
TGTACTCCCTGCTGTTCCTGCTGGGTCTGCTGGGTAACGGTGCTGTGGCTGCTGTGCTGCTGTCTA
GAAGAACTGCTCTGTCCTCCACTGACACTTTCCTGCTGCACCTGGCTGTGGCTGACACTCTGCTGG

TGCTGACTCTGCCACTGTGGGCTGTGGACGCTGCTGTGCAGTGGGTGTTCGGTTCCGGTCTGTGC
AAGGTGGCTGGTGCTTTGTTCAACATCAACTTCTACGCTGGTGCTCTGTTGCTGGCTTGCATCTCCT
TCGACAGATACCTGAACATCGTGCACGCTACTCAGCTGTACAGAAGAGGTCCACCTGCTAGAGTGA
CTCTGACTTGCCTGGCTGTGTGGGGTCTGTGCCTGCTGTTCGCTCTGCCTGACTTCATCTTCCTGT
CCGCTCACCACGACGAGAGACTGAACGCTACTCACTGTCAGTACAACTTCCCACAAGTGGGTCGC
ACTGCTCTGAGAGTGCTGCAGCTGGTGGCTGGTTTCCTGCTGCCACTGCTGGTGATGGCTTACTG
CTACGCTCACATCCTGGCTAGACTGAAGTCCGTGAGACTGCTGTCCGGTTCTAGAGAGAAGGACA
GAAACCTGAGAAGAATCACTAGACTGGTGGTCGTGGTCGTGGTGGCTTTTGCTCTGTGCTGGACTC
CATACCACCTGGTCGTGCTGGTGGACATCCTGATGGACCTGGGTGCTCTGGCTAGAAACTGCGGT
AGAGAGTCTAGAGTGGACGTGGCTAAGTCCGTGACTTCCGGTCTGGGTTACATGCACTGCTGCCT
GAACCCACTGCTGTACGCTTTCGTGGGTGTGAAGTTCAGAGAGAGAATGTGGATGTTGCTGCTGC
GCCTGGGTTGCCCAAATCAGAGAGGTCTGCAGAGACAGCCATCCTCTTCTAGAAGAGACTCCTCCT
GGTCCGAGACTTCCTAA

CXCL11 (in pFaseBac1)

ATGAGTGTGAAGGGCATGGCTATAGCCTTGGCTGTGATATTGTGTGCTACAGTTGTTCAAGGCTTCC
CCATGTTCAAAAGAGGACGCTGTCTTTGCATAGGCCCTGGGGTAAAAGCAGTGAAAGTGGCAGATA
TTGAGAAAGCCTCCATAATGTACCCAAGTAACAACTGTGACAAAATAGAAGTGATTATTACCCTGAAA
GAAAATAAAGGACAACGATGCCTAAATCCCAAATCGAAGCAAGCAAGGCTTATAATCAAAAAAGTTGA
AAGAAAGAATTTTcaccatcaccatcaccatcaccatcaccatTAG

Supplementary Table 2. Primers for the generation of CXCR3 and its mutants used in cAMP assay.

Construct	Vector	Primers
CXCR3	pcDNA3.1	F: CTGTTCCAGGGGCCCGGATCCATGGTGCTGGAGGTGTCCGAC<br>R: GATGGTGATGCTCGAGTCACAAGCCCGAGTAGGAGGCCTCTGAAGTCTCGGACCAGGAG
CXCR3-D52A	pcDNA3.1	F: GACTTCTCCCTGAACTTCGCTAGAGCTTTCCTGCCTGCTC<br>R: GAGCAGGCAGGAAAGCTCTAGCGAAGTTCAGGGAGAAGTC
CXCR3-Y60A	pcDNA3.1	F: GCTTTCCTGCCTGCTCTGGCTTCCCTGCTGTTCCTGCTGG<br>R: CCAGCAGGAACAGCAGGGAAGCCAGAGCAGGCAGGAAAGC
CXCR3-W109A	pcDNA3.1	F: GTGCTGACTCTGCCACTGGCTGCTGTGGACGCTGCTGTGC<br>R: GCACAGCAGCGTCCACAGCAGCCAGTGGCAGAGTCAGCAC
CXCR3-F131A	pcDNA3.1	F: GCAAGGTGGCTGGTGCTTTGGCTAACATCAACTTCTACGCTG<br>R: CAGCGTAGAAGTTGATGTTAGCCAAAGCACCAGCCACCTTGC
CXCR3-F135A	pcDNA3.1	F: GCTTTGTTCAACATCAACGCTTACGCTGGTGCTCTGTTGC<br>R: GCAACAGAGCACCAGCGTAAGCGTTGATGTTGAACAAAGC
CXCR3-R197A	pcDNA3.1	F: TCCGCTCACCACGACGAGGCTCTGAACGCTACTCACTGTC<br>R: GACAGTGAGTAGCGTTCAGAGCCTCGTCGTGGTGAGCGGA
CXCR3-R212A	pcDNA3.1	F: CAACTTCCCACAAGTGGGTGCTACTGCTCTGAGAGTGCTGCAG<br>R: CTGCAGCACTCTCAGAGCAGTAGCACCCACTTGTGGGAAGTTG
CXCR3-W268A	pcDNA3.1	F: GTGGCTTTTGCTCTGTGCGCTACTCCATACCACCTGGTCG<br>R: CGACCAGGTGGTATGGAGTAGCGCACAGAGCAAAAGCCAC
CXCR3-Y271A	pcDNA3.1	F: GCTCTGTGCTGGACTCCAGCTCACCTGGTCGTGCTGGTGG<br>R: CCACCAGCACGACCAGGTGAGCTGGAGTCCAGCACAGAGC
CXCR3-D282A	pcDNA3.1	F: CTGGTGGACATCCTGATGGCTCTGGGTGCTCTGGCTAGAA<br>R: TTCTAGCCAGAGCACCCAGAGCCATCAGGATGTCCACCAG
CXCR3-E293A	pcDNA3.1	F: GCTAGAAACTGCGGTAGAGCTTCTAGAGTGGACGTGGCTA<br>R: TAGCCACGTCCACTCTAGAAGCTCTACCGCAGTTTCTAGC
CXCR3-S304L	pcDNA3.1	F: CTAAGTCCGTGACTTTGGGTCTGGGTTACATGC<br>R: GCATGTAACCCAGACCCAAAGTCACGGACTTAG
CXCR3-G307A	pcDNA3.1	F: TCCGTGACTTCCGGTCTGGCTTACATGCACTGCTGCCTGA<br>R: TCAGGCAGCAGTGCATGTAAGCCAGACCGGAAGTCACGGA
CXCR3-Y308A	pcDNA3.1	F: GTGACTTCCGGTCTGGGTGCTATGCACTGCTGCCTGAACC<br>R: GGTTCAGGCAGCAGTGCATAGCACCCAGACCGGAAGTCAC
CXCR3-A265F	pcDNA3.1	F: GTCGTGGTGGCTTTTTTTCTGTGCTGGACTC<br>R: GAGTCCAGCACAGAAAAAAAGCCACCACGAC
CXCR3-V261F	pcDNA3.1	F: CTGGTGGTCGTGGTCTTCGTGGCTTTTGCTCTG<br>R: CAGAGCAAAAGCCACGAAGACCACGACCACCAG
