# Supplementary material for: Structural insights into the activation and inhibition of CXC chemokine receptor 3
Source: Nat Struct Mol Biol. 2024 Jan 4;31(4):610–20. doi: 10.1038/s41594-023-01175-5 (PMC11026165; doi:10.1038/s41594-023-01175-5)
Supplement: Supplementary file 2 — Reporting Summary [file 41594_2023_1175_MOESM2_ESM.pdf]

nature portfolio

Corresponding author(s): HU Hongli

Last updated by author(s): Oct 2, 2023

Reporting Summary

Nature Portfolio wishes to improve the reproducibility of the work that we publish. This form provides structure for consistency and transparency in reporting. For further information on Nature Portfolio policies, see our Editorial Policies and the Editorial Policy Checklist.

Statistics

For all statistical analyses, confirm that the following items are present in the figure legend, table legend, main text, or Methods section.

n/a	Confirmed	
☐	☒	The exact sample size (*n*) for each experimental group/condition, given as a discrete number and unit of measurement
☐	☒	A statement on whether measurements were taken from distinct samples or whether the same sample was measured repeatedly
☒	☐	The statistical test(s) used AND whether they are one- or two-sided<br>*Only common tests should be described solely by name; describe more complex techniques in the Methods section.*
☒	☐	A description of all covariates tested
☒	☐	A description of any assumptions or corrections, such as tests of normality and adjustment for multiple comparisons
☐	☒	A full description of the statistical parameters including central tendency (e.g. means) or other basic estimates (e.g. regression coefficient) AND variation (e.g. standard deviation) or associated estimates of uncertainty (e.g. confidence intervals)
☒	☐	For null hypothesis testing, the test statistic (e.g. *F*, *t*, *r*) with confidence intervals, effect sizes, degrees of freedom and *P* value noted<br>*Give P values as exact values whenever suitable.*
☒	☐	For Bayesian analysis, information on the choice of priors and Markov chain Monte Carlo settings
☒	☐	For hierarchical and complex designs, identification of the appropriate level for tests and full reporting of outcomes
☒	☐	Estimates of effect sizes (e.g. Cohen's *d*, Pearson's *r*), indicating how they were calculated

Our web collection on statistics for biologists contains articles on many of the points above.

Software and code

Policy information about availability of computer code

Data collection	SerialEM 3.8
Data analysis	MotionCor2, Gctf 1.18, RELION 3.1, CryoSPARC 3.3.1, UCSF ChimeraX 1.4, Coot 0.9.8.1, PyMOL 2.0, Phenix 1.20.1, CCP-EM 1.6.0, AceDRG 246, LocScale, GraphPad Prism 8.0, CHARMM-GUI 3.8, Gromacs 2021.4.

For manuscripts utilizing custom algorithms or software that are central to the research but not yet described in published literature, software must be made available to editors and reviewers. We strongly encourage code deposition in a community repository (e.g. GitHub). See the Nature Portfolio guidelines for submitting code & software for further information.

Data

Policy information about availability of data

All manuscripts must include a data availability statement. This statement should provide the following information, where applicable:

- Accession codes, unique identifiers, or web links for publicly available datasets
- A description of any restrictions on data availability
- For clinical datasets or third party data, please ensure that the statement adheres to our policy

The cryo-EM density maps generated in this study have been deposited in Electron Microscopy Data Bank under accession codes: EMD-34914 (CXCR3-CXCL11-DNGi-scFv16), EMD-34915 (CXCR3-PS372424-DNGi-scFv16), EMD-34916 (CXCR3-VUF11222-DNGi-scFv16), and EMD-34917 (CXCR3κOR-SCH546738-Nb6). The

associated protein models have been deposited in the Protein Data Bank under accession codes: 8HNK (CXCR3-CXCL11-DNGi-scFv16), 8HNL (CXCR3-PS372424-DNGi-scFv16), 8HNM (CXCR3-VUF11222-DNGi-scFv16), and 8HNN (CXCR3κOR-SCH546738-Nb6). Source data are provided with this paper.

Research involving human participants, their data, or biological material

Policy information about studies with human participants or human data. See also policy information about sex, gender (identity/presentation), and sexual orientation and race, ethnicity and racism.

Reporting on sex and gender	N/A
Reporting on race, ethnicity, or other socially relevant groupings	N/A
Population characteristics	N/A
Recruitment	N/A
Ethics oversight	N/A

Note that full information on the approval of the study protocol must also be provided in the manuscript.

Field-specific reporting

Please select the one below that is the best fit for your research. If you are not sure, read the appropriate sections before making your selection.

☒ Life sciences ☐ Behavioural & social sciences ☐ Ecological, evolutionary & environmental sciences

For a reference copy of the document with all sections, see nature.com/documents/nr-reporting-summary-flat.pdf

Life sciences study design

All studies must disclose on these points even when the disclosure is negative.

Sample size	No sample calculation was performed for the cryo-EM analysis. The sample size of cryo-EM micrographs was determined by the availability of the microscope time. The cryo-EM micrographs collected are: 5500 (CXCR3-CXCL11-DNGi-scFv16), 3185 (CXCR3-PS372424-DNGi-scFv16), 2891 (CXCR3-VUF11222-DNGi-scFv16), and 12944 (CXCR3κOR-SCH546738-Nb6). The sample size of particles used in the final reconstruction was determined by reported resolution and the quality of the density map. The final particles used for cryo-EM reconstruction are: 96877 (CXCR3-CXCL11-DNGi-scFv16), 389182 (CXCR3-PS372424-DNGi-scFv16), 162856 (CXCR3-VUF11222-DNGi-scFv16), and 509297 (CXCR3κOR-SCH546738-Nb6).<br>For cAMP assay, 3 to 6 independent experiments were performed as indicated in the figure legends.
Data exclusions	During data processing, the particles with bad alignment were excluded from the final reconstruction based on the 2D averages or 3D classification map as implemented in RELION and CryoSPARC.
Replication	For cryo-EM, no replication studies were attempted. The primary data is cryo-EM structures that are calculated according to standard procedures and do not need replicates. A replication of the cryo-EM data collection with the same sample size was not economically justifiable and the time cost was high.<br>For cAMP assay, each experiment was replicated at least twice on separate occasions.
Randomization	Randomization is irrelevant for our study because no grouping was needed. For single-particle cryo-EM analysis, particles are divided into two random sets for map reconstruction and estimation of the resolution via the Fourier Shell Correlation (FSC) method.
Blinding	Blinding is irrelevant to our study because no allocation into experimental groups was needed.

Reporting for specific materials, systems and methods

We require information from authors about some types of materials, experimental systems and methods used in many studies. Here, indicate whether each material, system or method listed is relevant to your study. If you are not sure if a list item applies to your research, read the appropriate section before selecting a response.

Materials & experimental systems

n/a	Involved in the study
☐	☒ Antibodies
☐	☒ Eukaryotic cell lines
☒	☐ Palaeontology and archaeology
☒	☐ Animals and other organisms
☒	☐ Clinical data
☒	☐ Dual use research of concern
☒	☐ Plants

Methods

n/a	Involved in the study
☒	☐ ChIP-seq
☒	☐ Flow cytometry
☒	☐ MRI-based neuroimaging

Antibodies

Antibodies used	Anti Flag-Tag Mouse Monoclonal Antibody (CWBIO, Cat# CW0287), clone name F-tag-01<br>Alexa Fluor 488-labeled Goat Anti-Mouse IgG (Beyotime, Cat# A0428)
Validation	Anti Flag-Tag Mouse Monoclonal Antibody (CWBIO, Cat# CW0287)<br>Validation statement from manufacturer's website: The antibody is highly specific to recognize the Flag tag at the C-terminal or N-terminal of the recombinant protein without being affected by neighboring amino acids, and can be used to detect Flag-tag fusion proteins expressed by various expression vectors. Clone antibody type: rat monoclonal antibody. IgG number: F-tag-01. Immunogen: synthetic peptide (DYKDDDDK). Antibody concentration: 0.5 mg/ml. Application: WB (1:500-5000), IP (1:50-200), IF/ICC, ELISA.<br>Alexa Fluor 488-labeled Goat Anti-Mouse IgG (Beyotime, Cat# A0428)<br>Validation statement from manufacturer's website: This Alexa Fluor 488-labeled Goat Anti-Mouse IgG(H+L) could be used for immunofluorescence staining. The antibody is produced by immuning goats with purified mouse IgG, and has little binding ability to human IgG, horse IgG, bovine IgG(bovine IgG), rabbit IgG and pig IgG. It is especially suitable for fluorescent staining experiment which requires high specificity of secondary antibody species. This Alexa Fluor 488-labeled Goat anti-mouse IgG(H+L) has a recommended dilution ratio of 1:500 for immunofluorescence staining.

Eukaryotic cell lines

Policy information about cell lines and Sex and Gender in Research

Cell line source(s)	Sf9 (Thermo Fisher Scientific, Cat# 11496015)<br>HEK293T (ATCC, Cat# CRL-11268)
Authentication	None of the cell lines used were authenticated.
Mycoplasma contamination	Cell lines were not tested for mycoplasma contamination.
Commonly misidentified lines<br>(See ICLAC register)	No commonly misidentified cell lines were used in the study.
