# Supplementary figures and images for: Structural insights into the activation and inhibition of CXC chemokine receptor 3
Source: Nat Struct Mol Biol. 2024 Jan 4;31(4):610–20. doi: 10.1038/s41594-023-01175-5 (PMC11026165; doi:10.1038/s41594-023-01175-5)

Extended Data Fig. 1b

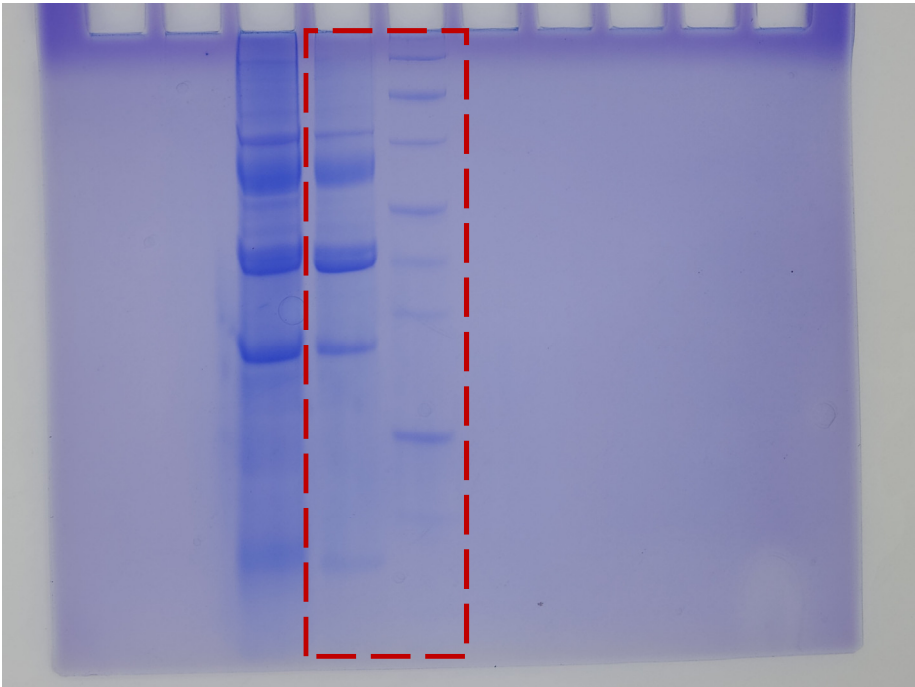

Extended Data Fig. 1e

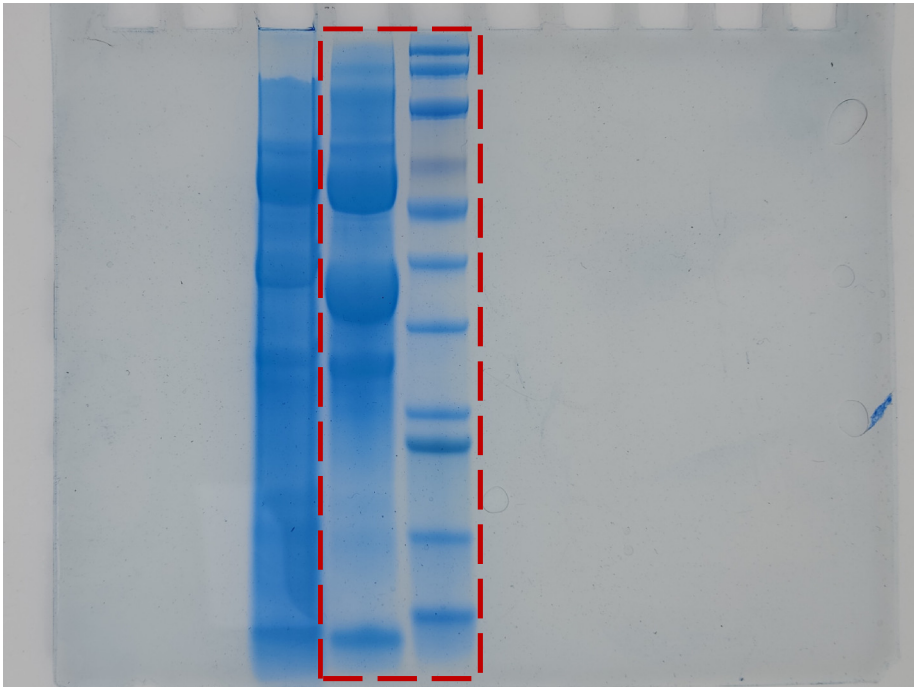

Extended Data Fig. 1h

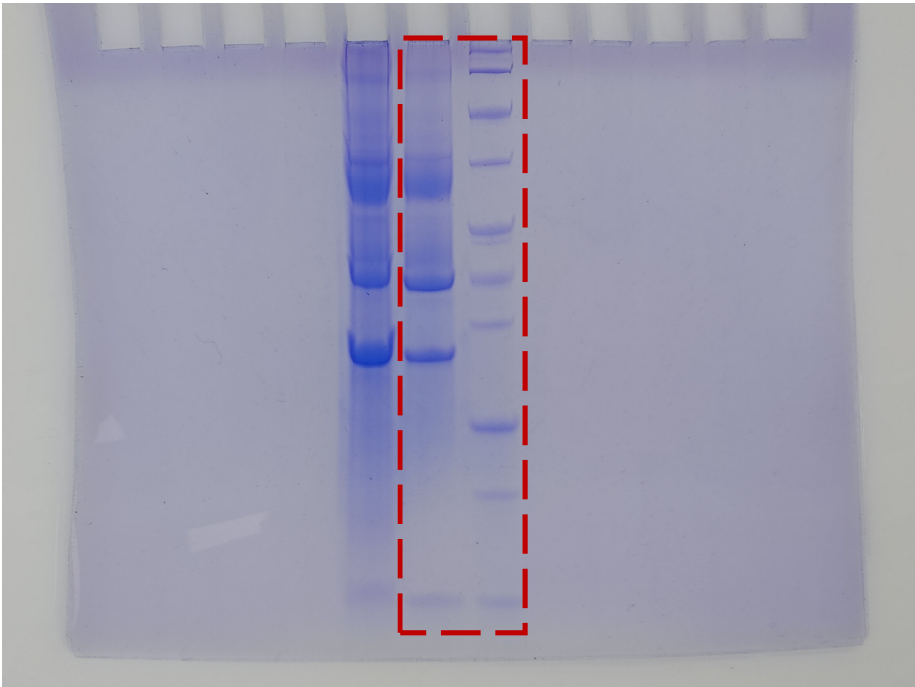

Extended Data Fig. 1k

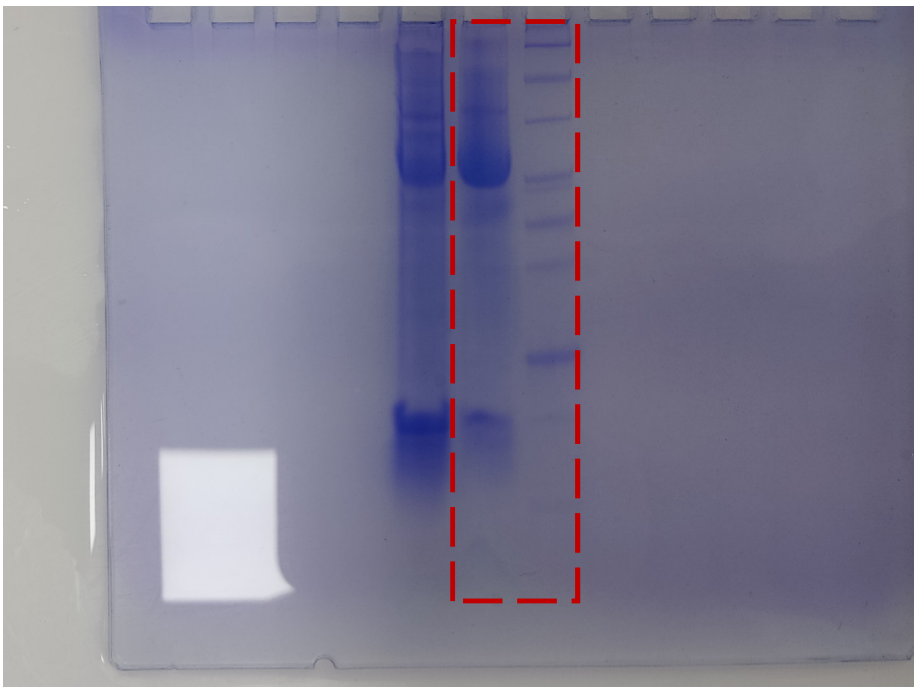

Supplement: Supplementary file 8 — Unprocessed gels. [file 41594_2023_1175_MOESM8_ESM.pdf]
